# Eye tracking for classification of concussion in adults and pediatrics

**DOI:** 10.3389/fneur.2022.1039955

**Published:** 2022-12-01

**Authors:** Uzma Samadani, Robert J. Spinner, Gerard Dynkowski, Susan Kirelik, Tory Schaaf, Stephen P. Wall, Paul Huang

**Affiliations:** ^1^Minneapolis Veterans Administration Health Care System, Minneapolis, MN, United States; ^2^Mayo Clinic, Rochester, MN, United States; ^3^Beaver Dam Community Hospital, Beaver Dam, WI, United States; ^4^Rocky Mountain Pediatric OrthoONE Center for Concussion, Denver, CO, United States; ^5^Rocky Mountain Hospital for Children, Denver, CO, United States; ^6^Ronald O. Perelman Department of Emergency Medicine and Department of Population Health, NYU Grossman School of Medicine, New York City, NY, United States; ^7^Bellevue Hospital and New York University Department of Neurosurgery, New York City, NY, United States

**Keywords:** concussion, eye movement tracking, baseline concussion, baseline, eye tracking

## Abstract

**Introduction:**

In order to obtain FDA Marketing Authorization for aid in the diagnosis of concussion, an eye tracking study in an intended use population was conducted.

**Methods:**

Potentially concussed subjects recruited in emergency department and concussion clinic settings prospectively underwent eye tracking and a subset of the Sport Concussion Assessment Tool 3 at 6 sites. The results of an eye tracking-based classifier model were then validated against a pre-specified algorithm with a cutoff for concussed vs. non-concussed. The sensitivity and specificity of eye tracking were calculated after plotting of the receiver operating characteristic curve and calculation of the AUC (area under curve).

**Results:**

When concussion is defined by SCAT3 subsets, the sensitivity and specificity of an eye tracking algorithm was 80.4 and 66.1%, The AUC was 0.718. The misclassification rate (*n* = 282) was 31.6%.

**Conclusion:**

A pre-specified algorithm and cutoff for diagnosis of concussion vs. non-concussion has a sensitivity and specificity that is useful as a baseline-free aid in diagnosis of concussion. Eye tracking has potential to serve as an objective “gold-standard” for detection of neurophysiologic disruption due to brain injury.

## Introduction

Traumatic brain injury (often abbreviated as TBI) is a multifaceted disease that may result in any of over 21 symptoms ranging from dizziness to photophobia (1). Current assessments for concussion, the most common type of TBI, rely on combining tests designed to evaluate functional domains known to be affected such as balance, memory, and cognition (2). An individual test for TBI may not be effective in diagnosing a patient if the test has not been designed to assess the specific symptoms with which the patient is presenting (3).

Because of the heterogeneity of the normal spectrum of brain function, many concussion detection tests require baseline assessments (4). Children with poorer academic achievement scores may do worse on these tests, particularly when reading is required (5). Tests requiring baselines are particularly vulnerable to developmental influence, learning curves, practice effect (6) and volitional exaggeration (7, 8). History of treatment for headaches, migraines, a psychiatric condition, and diagnosis of ADHD have been shown to affect baseline ImPACT scores (9). A baseline-free concussion diagnostic eliminates the possibility of “gaming” the test by failing or memorizing aspects of the baseline test, which occurs amongst athletes and military personnel, and reduces the amount of time required by athletic trainers and military personnel to conduct lengthy baseline testing sessions.

In this study only subsets of the Sports Concussion Assessment Tool 3 (SCAT3) symptom severity score (SSS) and standardized assessment of concussion (SAC) were used to define concussion. Version 3 of the SCAT was available at the time of the study. These subsets of SCAT3 are more appropriate for non-athletes than the complete SCAT3. Specifically, the following sections of the SCAT3 were not included for the accompanying reasons.

The Glasgow coma scale (GCS) was not used because any patient with a GCS below 14 should potentially be evaluated for more severe brain injury, and not enrolled in this clinical trial, e.g., eyes not opening spontaneously, no verbal response, no motor response. Some studies suggest that the GCS is not sensitive to less severe TBIs (10, 11).

The Maddocks Score section of the SCAT3 asks questions specific to the game the athlete is currently actively participating in and is therefore not appropriate for the emergency room or clinic setting (10, 11).

The balance examination is appropriate for athletes who are in training and normally have good to excellent balance. Unlike athletes, the general emergency department population often has comorbidities that preclude balance assessment, or are not meaningful without a baseline for comparison (10, 12).

For the purposes of assessing the eye movement-based algorithm for concussion, we defined concussion as (1) in the presence of AOC, SAC <23 and SSS >25, or (2) in the absence of AOC, subjects who exhibited SSS >32 and SAC <15.

An initial algorithm development study and preliminary pilot study of the BOX score algorithm have been reported by Samadani et al. and summarized (13, 14). The eye movement tracking algorithm detects disruption of central nervous system function and is sensitive for detection of acute mass effect in the brain (15). The algorithm also detects disruption of pathways controlling eye movements associated with structural TBI and concussion (16). Eye tracking is performed while a subject watches television or a video moving inside an aperture with a set trajectory for 220 s at a fixed distance from a viewing monitor. The position of each pupil is recorded over time elapsed as the video travels on its time course, enabling detection of impaired ability to rotate the eyes relative to time and therefore relative to each other. In previous work, it was demonstrated that the severity of disconjugate gaze in Emergency Department (ED) structural TBI and concussion patients detectable with this algorithm was proportionate to the severity of concussion symptoms.

The purpose of the current study is to validate the sensitivity and specificity of a baseline-free eye movement tracking algorithm developed by Samadani et al. (13) as a classifier for identifying concussion. The sensitivity and specificity of a baseline-free eye movement tracking based algorithm for concussion indicates that it is a useful aid in diagnosis of concussion in patients <2 weeks from injury. Eye tracking has potential to serve as a gold-standard for detection of physiologic disruption after brain injury.

## Methods

Patients aged 4–67 suspected of having a concussion were recruited from an Emergency Room or Concussion clinic at 6 independent sites. 282 subjects (177 adults and 105 pediatric patients) were successfully enrolled in the study. Eye tracking while watching a short film clip for 220 seconds, alteration of consciousness (AOC), and Sports Concussion Assessment Tool (SCAT3) subsets were collected (16, 17). The SCAT subsets were the symptom severity score (SSS) and standardized assessment of concussion (SAC). The patient's medical history was also obtained.

Alteration of consciousness was defined as documentation in the medical record by the clinicians caring for the patient that the patient had loss of consciousness, or was unresponsive or less responsive at any point after injury.

### Subject selection

Inclusion criteria for subjects were: able to provide written informed consent, ages between 4 and 67 years old (inclusive), have a suspected diagnosis of traumatic brain injury with a potential for concussion, have baseline vision correctable to within 20/500 bilaterally, have no prior history of diagnosed ocular motility disorder, and have the ability to provide a complete ophthalmologic, medical and neurologic history as well as medications/drugs/alcohol consumed within the 24 h prior to tracking. Exclusion criteria for patients were: injury which may have caused the concussion more than 2 years prior to enrollment, penetrating trauma, a head CT demonstrating evidence of acute brain injury (subdural, epidural or intraparenchymal hemorrhage, edema/mass effect per attending radiologist read), concurrent burn, anoxic injury or multiple/extensive injuries resulting in any medical, surgical or hemodynamic instability, blindness (no light perception), have missing eyes, be unable to open their eyes, have a prior history of ocular motility dysfunction, have had extensive prior eye surgery, have any physical or mental injury or baseline disability rendering task completion difficult, be intoxicated or have blood alcohol level >0.2, be a prisoner or in the company of a police officer or law enforcement officer pending arrest. The 2 year criteria for exclusion for previous concussion was set to exclude compounding effects from multiple head traumas.

This study was started in 2013, hence the older version of SCAT was utilized rather than updated versions. SCAT3 assessments were administered at the time of eye tracking by research personnel blinded to the eye tracking findings in patients.

For the purposes of assessing the eye tracking-based algorithm for concussion, we arbitrarily defined concussion as 1) in the presence of AOC, SAC < 23 and SSS > 25, or 2) in the absence of AOC, subjects who exhibited SSS> 32 and SAC < 15 (Note: Only 1 subject was in the latter group). Subjects meeting these criteria, and with a BOX Score ≥10 were considered “true positives” for concussion.

This work was conducted in order to obtain data for FDA marketing authorization and therefore it was tested in an “intended use” population that included a mix of both children and adults. There were no control groups included because the intended use of the device was not in people who did not have a history of possible brain trauma. The study design was prospective and observational without randomization or inclusion of control groups. The research teams conducting the eye tracking were blinded to the clinical examination findings and the clinical teams were blinded to the research findings.

### Visual stimulus

We recorded subjects' eye movements using only a Eyelink 1000 eye tracker at a fixed distance of 55 cm from a computer monitor over a time period of 220 s. The Eyelink device is a stand alone device that allows for unassisted use. The distance was fixed by means of a chinrest attached to the base of the viewing monitor and camera. Subjects were seated in either a height adjustable or height-fixed chair or bed, with the monitor height adjusted to the subject as described previously (16). The visual stimuli were music or film video clips. The video was played continuously in a square aperture with an area ~1/8 the screen size while moving clockwise along the outer edges of a rectangular (aspect ratio 4:3) viewing monitor at a rate of 10 s per side for five complete cycles of 40 s each. The total visible span of the moving aperture was ~17° horizontally and 13 degrees vertically from midposition with a caveat that the subject may be viewing different portions of the aperture during each cycle. The first and last 10 s of each data set were discarded to yield 200 s of data. The afferent stimulus was presented binocularly and eye tracking was performed binocularly. Subjects were not spatially calibrated to the tracker to enable independent analysis of each pupil position over time. The presented eye tracking stimulus is not affected by confounding bias such as a disinterest or affinity toward the stimulus because multiple stimuli are presented and the ability for the eyes to track the motion is recorded.

### Data analysis

The eye tracker sampled pupil position at 500 Hz, yielding 100,000 samples over 200 s. We created scatterplots of the entire time series by plotting the 100,000 (*x, y*) pairs representing the two orthogonal components of the pupil position estimated by pupil-cornea reflection measurement over time to create ‘box trajectories' that reflected the temporal nature of the pupillary movement. These figures look like boxes, reflecting the timing of the aperture as it moved around the screen with each 10 s of data collection representing one unit of ocular traverse. Horizontally the pupil traveled ~34° over 10 s and vertically it traveled ~23° in 10 s. 200 data points prior to and following each blink were removed before creating the measures of disconjugacy and aspect ratio to limit noise in the data from the blink event.

Typical eye tracking experiments feature a gaze-point-fixation-based calibration system to train the eye tracker's internal model to be able to accurately predict the subject's gaze position on the screen. The baseline-free eye movement tracking algorithm used for this study is not training a model eye gaze model nor is it concerned about the accurate localization of gaze on a screen (18). Raw pupil coordinates from the EyeLink device were transformed based on values from each eye respectively, not mixing values across eyes, consistent with our assumption that brain injured patients have eyes that may not move together.

### Statistical analysis

Data were analyzed for “True Positives”, “True Negatives”, “False Positives” and “False Negatives” for the algorithm (BOX) score as compared with the clinical reference standard.

## Results

We tested the algorithm performance in a validation dataset consisting of 282 subjects.

The average age of the enrolled population (*n* = 282), with concussed and non-concussed according to the clinical refence standard is presented in Table 1. 177 adults, 72 pediatrics (ages 12–21 years) and 33 children (ages 5–11) were enrolled.

**Table 1:** Average age of the enrolled population and subsets.

	**Overall**	**Concussed**	**Non-concussed**
Average age (*N =* 282)	31.37 (SD = 17.1), range 5–67	33.43 (*N =* 46)	30.96 (*N =* 236)
Average age, adults (*N =* 177)	41.76 (SD = 12.91), range 22–67	44.6 (*N =* 29)	41.2 (*N =* 148)
Average age, pediatrics (12–21) (*N =* 72)	15.99 (SD = 2.27), range 13 – 21	17.57 (*N =* 11)	15.71 (*N =* 61)
Average age, child (5–11) (*N =* 33)	9.16 (SD = 1.89), range 5–12	8.50 (*N =* 6)	9.31 (*N =* 27)

## Algorithm validation

**Figure 1:**
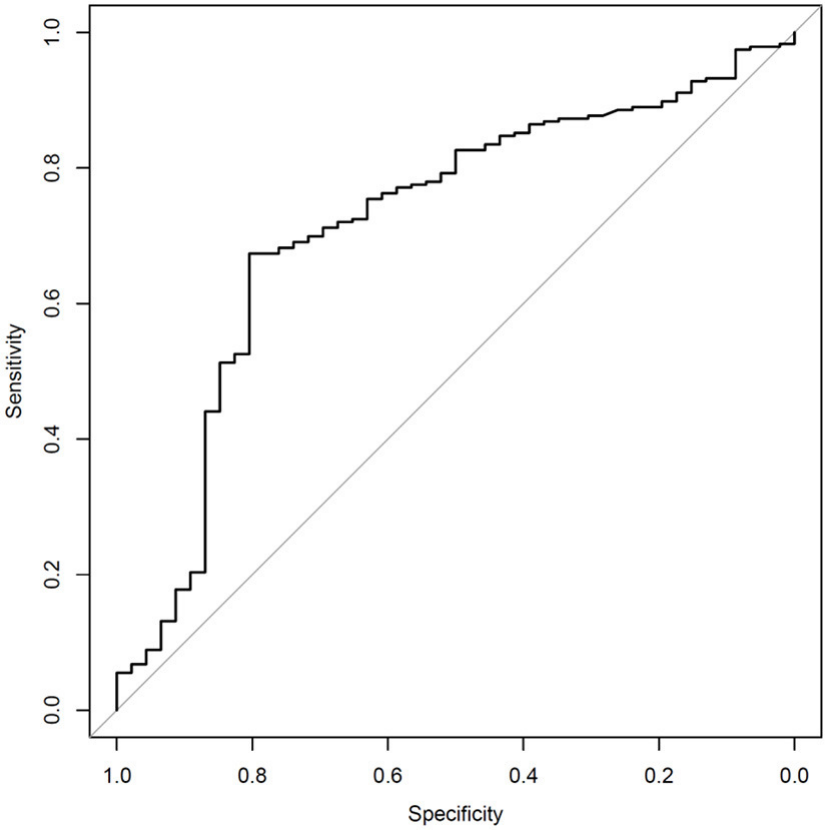
Receiver operating curve (ROC). AUC = 0.717.

The sensitivity and specificity of the algorithm are shown in Table 2. The receiver operating curve (ROC) is shown in Figure 1, has an AUC of 0.717. The numbers of true-positives (TP), false positives (FP), false negatives (FN), and true negatives (TN), are shown in Table 3. An analysis which excludes subjects with high neck pain, but low overall symptoms, from the *N* = 282 full data set was conducted per the following criteria to differentiate cervical injury symptoms from brain injury (17): Subjects with a high neck pain score of 5 or 6 (on a scale of 0 to 6), a score of 0 for nausea (on a scale of 0–6), and an average score of ≤4 on all symptoms were excluded for the below analysis. Based on the preceding criteria, 9 subjects were identified as “primary complaint of neck pain” subjects. Excluding these 9 patients from the study population resulted in 273 patients. Patients ages 12 or under are not asked about neck pain in the SCAT3 questionnaire. The SCAT3 reference test diagnosed 5 of these 9 “primary complaint of neck pain” subjects as concussed and 4 as non-concussed. The SCAT3 was used before 2016 instead of the newer SCAT5, that became available post 2016. The post concussion severity score used in both version is the same, so this should not alter the outcome. When excluding subjects with a primary neck pain diagnosis, the sensitivity improves to 83.7% and the specificity remains approximately the same at 65.6%.

**Table 2:** Sensitivity and specificity of the algorithm (BOX Score) vs. the clinical reference standard.

Sensitivity	80.40%
95% CI	69.0–91.9%
Specificity	66.10%
95% CI	60.1–72.1%

**Table 3:** TP, FP, FN and TN values.

	**Concussed**	**Non-concussed**
Algorithm (BOX score) positive results	TP = 47	FP = 70
Algorithm (BOX score) negative results	FN *=* 20	TN *=* 145

## Discussion

Concussion is challenging to diagnose because many of the functions it disrupts are difficult to measure objectively. Many current tools available to assess concussion could be impacted by factors other than the concussion such as interest, hunger, fatigue, distractibility, education level and cultural factors. The purpose of this work is to determine the sensitivity and specificity of a baseline-free objective eye movement tracking algorithm for acute concussion. Diagnostics for traumatic brain injury include multiple forms of physical and cognitive assessment, imaging, electrical, cellular/molecular markers, and physiologic assessment. Heterogeneity of baseline function, variability in assessor experience and skill, and volitional behaviors render classification of the nature of injury difficult. Eye movements are reflexively coordinated in the brainstem with inputs from multiple cortical and subcortical tracts and thus are an ideal physiology to examine as an objective measure of dysfunction.

The eye movement tracking algorithm assessed in this work, referred to subsequently as the EyeBOX test, provides an objective measure of eye tracking abnormalities to augment the current methods of evaluation of the patient with suspected concussion. The reported sensitivity and specificity indicate consistency with an arbitrarily defined clinical reference standard of symptom severity plus cognitive assessment. Because there is currently no gold-standard clinical definition of concussion and no known cut-off value that is widely validated, we had to arbitrarily select a cutoff. In addition, the SCAT tests are designed for use in athletes rather than the general population, who have more diverse comorbidities. Because there are relatively few objective validated measures for concussion, an arbitrary cutoff had to be selected.

We would propose that the EyeBOX test is a purely objective measure of physiologic function that provides an additional dimension to concussion diagnosis in a heterogeneous population and would not be expected to duplicate the results of a clinical symptom assessment designed for athletes and based on a participant's subjective input, volitional effort and baseline cognitive reserve.

A widely used TBI diagnostic called the ImPACT test reported a sensitivity and specificity of 81.9 and 89.4%, respectively, in a 2006 study (19). One caveat in comparing the sensitivity and specificity of the ImPACT test to EyeBOX, is that ImPACT uses the same criteria to make its assessment as the criteria in their reference standard. In addition, the population studied for the ImPACT test is relatively homogenous group of high school athletes, not all of whom were injured. This increases the number of TN (true negatives)'s and thus the specificity of the test beyond what would occur in EyeBOX's intended use population, which only included trauma patients.

Our eye-tracking study confirms high sensitivity in a heterogenous population of emergency department and concussion clinic patients. This study was designed to specifically assess TBI across a broad range of ages and injuries in the injured intended use population. It is compared to an assessment designed for athletes primarily because relatively few other validated objective measures exist.

In summary, the present study enrolled a heterogeneous population presenting to the emergency department or concussion clinic (the age of subjects ranged from 5 to 67 years and gender was balanced). An eye tracking score, which is based on measures of ocular dysmotility, was compared to a combination of self-reported symptoms and a cognitive assessment and resulted in a sensitivity and specificity of 80.4 and 66.1%. The reason that this is an acceptable level of performance is that the data was taken from a heterogeneous population, which was compared to an independent reference standard without an uninjured control group to increase its specificity. The results from multiple eye tracking clinical trials and recent FDA approval of the EyeBox set the stage for a larger scale clinical trial to define how specific attributes or deficiency in eye-tracking ability are associated with symptoms, brain imaging, and outcomes. Future trials may include newly designed wearable eye tracking goggles, which will enhance the ease of use and increase accuracy and precision of measurement to identify individualized eye tracking fingerprints of brain injury.

## Data availability statement

The raw data supporting the conclusions of this article will be made available by the authors, without undue reservation.

## Ethics statement

The studies involving human participants were reviewed and approved by Bellevue IRB, Mayo Clinic IRB, Allegheny IRB and Western IRB. Written informed consent was obtained from all participants or their legal guardian/next of kin.

## Author contributions

All authors participated in reviewing the data and reporting the results.

## Funding

This study was partially funded by Oculogica and the device used is the company's EyeBOX. US has received funding from Abbott Diagnostic Laboratories, Hennepin County Medical Center, Hennepin Health Foundation, Integra Corporation, Steven and Alexandra Cohen Foundation for Veteran Post Traumatic Stress and Traumatic Brain Injury, United States Veterans Administration and Office of Research and Development, Minnesota State Office of Higher Education, J. Aron Allen Legacy Foundation, National Institutes of Health, Department of Defense, a Foundation created by the Friends and Family of Charlene Barron, by the family of Tim Healy, by the family of James and Patricia Andersen and by Traci Fernandez. In addition, US has intellectual property related to this work which is assigned to the United States Department of Veterans Affairs, New York University, and Hennepin County Medical Center and licensed to Oculogica Inc, a corporation in which US has equity and for which she serves on the board. Oculogica has received funding from National Space and Biomedical Research Institute, Department of Defense, National Institutes of Health, StartX, the New York City Economic Development Council, and New York University. US also has intellectual property for assessment of brain injury that is assigned to the United States Dept of Veterans Affairs, New York University, and Hennepin County Medical Center and not licensed. US has also served as a consultant, received speaker's honoraria, or been compensated for participation in review sections from Barbara Turnbull Foundation/BRAIN Canada; Google; Cottage Health; Minnesota Brain Injury Alliance; Minnesota, Texas, Louisiana, Wisconsin, Wyoming High School Coaches Association; National Football League; National Neurotrauma Society; North American Brain Injury Society; USA Football; Integra Corporation; and Medtronic. SW has received funding from the Insurance Institute for Highway Safety, NIH FIC, NIDDK, and NHLBI for research unrelated to that presented in this paper.

## Conflict of interest

Author US has an equity interest in the technology investigated in this paper *via* ownership of intellectual property assigned to NYU, VA, and HCMC and licensed to Oculogica Inc. The remaining authors declare that the research was conducted in the absence of any commercial or financial relationships that could be construed as a potential conflict of interest.

## Publisher's note

All claims expressed in this article are solely those of the authors and do not necessarily represent those of their affiliated organizations, or those of the publisher, the editors and the reviewers. Any product that may be evaluated in this article, or claim that may be made by its manufacturer, is not guaranteed or endorsed by the publisher.

## References

[1] Ellis MJ Cordingley D Vis S Reimer K Leiter J Russell K. Vestibulo-ocular dysfunction in pediatric sports-related concussion. J Neurosurg Pediatr. (2015) 16:248–55. 10.3171/2015.1.PEDS14524 26031619

[2] Gardner AJ Shih SL Adamov EV Zafonte RD. Research frontiers in traumatic brain injury: defining the injury. Phys Med Rehabil Clin N Am. (2017) 28:413–31. 10.1016/j.pmr.2016.12.014 28390522

[3] Borazjani R Ajdari MR Niakan A. Current Status and Outcomes of Critical Traumatic Brain Injury (GCS = 3-5) in a Developing Country: A Retrospective, Registry-Based Study. World J Surg. (2022) 46:2335–43. 10.1007/s00268-022-06645-3 35789431

[4] Lindgren KP Jaffe AE Kaysen D Teachman BA Young-McCaughan S Peterson AL. Implicit trauma identity associations in treatment-seeking U.S. military personnel do not predict or change in response to cognitive processing therapy for PTSD. Psychol Trauma. (2022). 10.1037/tra0001367 36174156 PMC10050228

[5] Krumholtz I. Results from a pediatric vision screening and its ability to predict academic performance. Optometry. (2000) 71:426–30. 15326895

[6] Valovich McLeod TC Perrin DH Guskiewicz KM Shultz SJ Diamond R Gansneder BM. Serial administration of clinical concussion assessments and learning effects in healthy young athletes. Controll Clin Trial Clin J Sport Med. (2004) 14:287–95. 10.1097/00042752-200409000-00007 15377968

[7] Cole WR Arrieux JP Schwab K Ivins BJ Qashu FM Lewis SC. Test-retest reliability of four computerized neurocognitive assessment tools in an active duty military population. Arch Clin Neuropsychol. (2013) 28:732–42. 10.1093/arclin/act040 23819991

[8] Resch J Driscoll A McCaffrey N. ImPact test-retest reliability: reliably unreliable? Journal of athletic training Jul-Aug. (2013) 48:506–11. 10.4085/1062-6050-48.3.09 23724770 PMC3718353

[9] Cottle JE Hall EE Patel K Barnes KP Ketcham CJ. Concussion baseline testing: preexisting factors, symptoms, and neurocognitive performance. J Athl Train. (2017) 52:77–81. 10.4085/1062-6050-51.12.21 28071936 PMC5343531

[10] Bin Zahid A Hubbard ME Dammavalam VM Balser DY Pierre G Kim A. Assessment of acute head injury in an emergency department population using sport concussion assessment tool–3rd edition. Appl Neuropsychol Adult. (2016) 25:110–9. 10.1080/23279095.2016.1248765 27854143

[11] Guskiewicz KM Register-Mihalik J McCrory P McCrea M Johnston K Makdissi M. Evidence-based approach to revising the SCAT2: Introducing the SCAT3. Br J Sports Med. (2013) 47:289–93. 10.1136/bjsports-2013-092225 23479486

[12] Kyle Harrold G Hasanaj L Moehringer N Zhang I Nolan R Serrano L. Rapid sideline performance meets outpatient clinic: Results from a multidisciplinary concussion center registry. J Neurol Sci. (2017) 379:312–7. 10.1016/j.jns.2017.06.038 28716270

[13] Samadani U Li M Qian M Laska E Ritlop R Kolecki R. Sensitivity and specificity of an eye movement tracking-based biomarker for concussion. Concussion. (2016) 1:1–14. 10.2217/cnc.15.2 30202548 PMC6114025

[14] Brett BL Kramer MD McCrea MA Broglio SP McAllister TW Nelson LD. Bifactor model of the sport concussion assessment tool symptom checklist: replication and invariance across time in the CARE consortium sample. Am J Sports Med. (2020) 48:2783–95. 10.1177/0363546520946056 32809856 PMC7484253

[15] Samadani U Farooq S Ritlop R Warren F Reyes M Lamm E. Detection of third and sixth cranial nerve palsies with a novel method for eye tracking while watching a short film clip. J Neurosurg. (2014) 122:707–20. 10.13070/ev.en.2.1366 25495739 PMC4547625

[16] Samadani U Ritlop R Reyes M Nehrbass E Li M Lamm E. Eye tracking detects disconjugate eye movements associated with structural traumatic brain injury and concussion. J Neurotrauma. (2015) 32:548–56. 10.1089/neu.2014.3687 25582436 PMC4394159

[17] Cheever K Kawata K Tierney R Galgon A. Cervical injury assessments for concussion evaluation: a review. J Athl Train. (2016) 51:1037–44. 10.4085/1062-6050-51.12.15 27835042 PMC5264559

[18] Bin Zahid A Hubbard ME Lockyer J Podolak O Dammavalam VM Grady M. Eye tracking as a biomarker for concussion in the pediatric population. Pending Submission (2016) 10.1097/JSM.0000000000000639 30095503

[19] Schatz P Putz BO. Cross-validation of measures used for computer-based assessment of concussion. Appl Neuropsychol. (2006) 13:151–9. 10.1207/s15324826an1303_2 17361667

